# Ischemic Postconditioning-Mediated miRNA-21 Protects against Cardiac ischemia/reperfusion Injury via PTEN/Akt Pathway

**DOI:** 10.1371/journal.pone.0075872

**Published:** 2013-10-03

**Authors:** Yingfeng Tu, Lin Wan, Yuhua Fan, Kezheng Wang, Lihong Bu, Tao Huang, Zhen Cheng, Baozhong Shen

**Affiliations:** 1 Radiology Department and Key Laboratory of Molecular Imaging, the Fourth Hospital of Harbin Medical University, Harbin, Heilongjiang, People’s Republic of China; 2 Department of Cardiology, the Fourth Hospital of Harbin Medical University, Harbin, Heilongjiang, People’s Republic of China; 3 Institute of Clinical Pharmacology of the Second Affiliated Hospital, Harbin Medical University, Harbin, Heilongjiang, People’s Republic of China; 4 Molecular Imaging Program at Stanford, Department of Radiology and Bio-X Program, Stanford University, Stanford, California, United States of America; Virginia Commonwealth University Medical center, United States of America

## Abstract

**Background:**

Ischemic postconditioning (IPost) protects the reperfused heart from infarction which has drawn much attention recently. However, studies to date have rarely investigated the role of microRNAs (miRNAs) in IPost. The aims of this study were to investigate whether miR-21 is involved in the protective effect of IPost against myocardial ischemia-reperfusion (I/R) injury and disclose the potential molecular mechanisms involved.

**Methods and Results:**

We found that miR-21 was remarkably up-regulated in mouse hearts after IPost. To determine the protective role of IPost-induced miR-21 up-regulation, the mice were divided into the following four groups: I/R group; I/R+IPost group (I/R mice treated with IPost); Antagomir-21+IPost+I/R group (I/R mice treated with anagomir-21 and IPost); Scramble+IPost+I/R group (I/R mice treated with scramble and IPost). The results showed IPost could reduce I/R injury-induced infarct size of the left ventricle, improve cardiac function, and prevent myocardial apoptosis, while knockdown of miR-21 with antagomir-21 could reverse these protective effects of IPost against mouse I/R injury. Furthermore, we confirmed that miR-21 plays a protective role in myocardial apoptosis through PTEN/Akt signaling pathway, which was abrogated by the PI3K inhibitor LY294002. The protective effect of miR-21 on myocardial apoptosis was further revealed in mouse hearts after IPost treatment in vivo.

**Conclusions:**

Our data clearly demonstrate that miR-21 is involved in IPost-mediated cardiac protection against I/R injury and dysfunction through the PTEN/Akt signaling pathway in vivo. Identifying the beneficial roles of IPost-regulated miRNAs in cardiac protection, which may be a rational target selection for ischemic cardioprotection.

## Introduction

Despite current optimal treatment, ischemic heart disease is still the leading cause of death all over the world [1]. The present normal treatment for myocardial ischemia is rapid reperfusion, which can attenuate myocardial infarction, reduce cardiomyocyte apoptosis and restore contractile dysfunction. Although timely reflow undoubtedly limits the extent of myocardial necrosis and then reduces mortality, numerous studies have demonstrated that reperfusion itself can initiate both transient and lethal injury following ischemia, i.e. ischemia/reperfusion (I/R) injury [2]. Ischemic preconditioning (IPC), as a powerful endogenous protective mechanism, has been demonstrated to reduce infarct size [3], diminish apoptosis [4], preserve vascular endothelial function [5], and prevent appearance of reperfusion arrhythmias [6]. Nevertheless, IPC itself is not a clinically practicable cardioprotective strategy because it has to be performed prior to myocardial infarction.

Ischemic postconditioning (IPost), defined as a short series of repetitive cycles of brief reperfusion and re-occlusion applied at the onset of reperfusion after a prolonged ischemic insult [7]. Recent studies in dogs [8], mice [9], rats [7], pigs [10], rabbits [11] and humans [12] have been reported that IPost plays a critical role in limiting infarct size, diminishing necrosis and apoptosis, improving vascular endothelial dysfunction, and preventing heart failure [13,14]. Unlike IPC, IPost, a protective stimulus administrated just before reperfusion, can be easily performed as a postischemic intervention to reduce the cellular damages inpatients receiving emergent percutaneous coronary intervention (PCI) [15]. However, the potential molecular mechanisms activated by IPost have not been fully disclosed.

MicroRNAs (miRNAs), a novel class of endogenous, noncoding, single-stranded RNAs, have emerged as a group of important regulators via degradation or translational inhibition of their target mRNAs [16]. Increasing evidences indicate that miRNAs are involved in the regulation of I/R injury [17,18]. For example, miR-320 was involved in the regulation of I/R injury and knockdown of endogenous miR-320 provided protection against I/R-induced cardiomyocyte apoptosis via antithetical regulation of Hsp20 [19]. Cheng et al. [20] demonstrated that miR-21 was up-regulated by IPC, which protected heart against I/R injury via anti-apoptosis through its target programmed cell death 4 (PDCD4). Moreover, IPC-mediated cardiac protection in rat heart was inhibited by knockdown of endogenous miR-21 expression [20]. Recently, He et al. demonstrated that cardiac miR-1 and miR-133 were significantly increased by IPost during reperfusion in an I/R injury rat model, indicating some miRNAs may be involved in the regulation of cardiac IPost during reperfusion [21]. However, the possible roles of miRNAs and the potential molecular mechanisms that regulate gene expression in myocardial IPost are far from fully elucidated.

In this study we unexpectedly found that miR-21 was remarkably up-regulated in myocardium by IPost in vivo. Knockdown of miR-21 with antagomir-21 could reverse the protective effects of IPost. This study indicated that manipulating the expression of miR-21 was involved in the protective effect of myocardial IPost.

## Methods

### Animals

Healthy adult male Kunming mice (25–30g) used in the current study were maintained in cages at room temperature (23 ± 1°C), with a constant humidity (55 ± 5%), and had free access to food and water. All experimental protocols were pre-approved by the Experimental Animal Ethic Committee of Harbin Medical University, China. Use of animals was confirmed with the Guide for the Care and Use of Laboratory Animals published by the US National Institutes of Health (NIH Publication No. 85–23, revised 1996).

### Cardiac I/R injury and IPost animal models

Kunming mice were anesthetized with pentobarbital sodium (50mg/kg ip) before endotracheal intubation. After anesthesia, the animals were placed in a supine position and surface leads were placed subcutaneously to record the electrocardiogram (ECG). All surgical procedures were carried out under aseptic conditions. A lateral thoracotomy (1.5cm incision between the third and fourth ribs) was performed to provide exposure of the left anterior descending coronary artery (LAD). A 7-0 nylon suture was placed around the LAD at 2 to 3 mm from the tip of the left auricle, and a nontraumatic balloon occluder was placed over the artery. Coronary occlusion was induced by inflating the balloon occluder. Sham-operated animals underwent the same procedure but the balloon occluder was not inflated. I/R injury was induced by inflating the balloon occluder for 30 min, followed by 3 h of reperfusion. IPost was achieved via four cycles of 30 sec reperfusion/30 sec ischemia (total time, 4 min) given at the end of 30 min coronary occlusion [22]. In order to inhibit the PTEN/Akt signaling pathway, we used the PI3K inhibitor LY294002 (LY, 15µmol/L) given for the first 15 min of reperfusion. Ischemia was confirmed by visual observation (cyanosis) and by observing S-T segment elevation and QRS widen on ECG.

### MiRNA extraction and quantitative real-time RT-PCR analysis

Briefly, total RNA from mouse cardiac ventricular myocytes was extracted using Trizol reagent according to the protocol of the manufacturer (Invitrogen). The concentration of extracted total RNA was quantified by ultraviolet spectrophotometer, the A260/A280 ratio needed to be about 1.8~2.0. One microgram of total RNA from each sample was used to generate cDNA by using M-MLV reverse transcriptase (Applied Biosystems) with special stem-loop primer for miRNAs. Then real-time quantitative PCR was performed to quantify the expression level of miR-1, miR-9, miR-15b, miR-21, miR-23a, miR-24, miR-26a, miR-27, miR-133a, miR-199a, miR-208, miR-214 and miR-499 with SYBR Green PCR Master Mix (Applied Biosystems) according to the manufacturer’s instructions. The qRT-PCR was performed on a ABI 7500 thermocycler (Applied Biosystems) for 40 cycles. The 2^-ΔΔCT^ relative quantification method was applied, and U6 was used as an internal control. The comparative threshold cycle method was used to calculate the relative gene expression. Primers for amplification of U6, miR-1, miR-9, miR-15b, miR-21, miR-23a, miR-24, miR-26a, miR-27, miR-133a, miR-199a, miR-208, miR-214 and miR-499 were listed in Table 1.

**Table 1:** Primers used for quantitative real-time RT-PCR.

**RT-primers**	
miR-24	5' GTCGTATCCAGTGCGTGTCGTGGAGTCGGCAATTGCACTGGATACGACCTGTTCC-3'
miR-1	5'-GTCGTATCCAGTGCGTGTCGTGGAGTCGGCAATTGCACTGGATACGACATCATA-3'
miR-133	5'-GTCGTATCCAGTGCGTGTCGTGGAGTCGGCAATTGCACTGGATACGACCAGCTG-3'
miR-23a	5'-GTCGTATCCAGTGCGTGTCGTGGAGTCGGCAATTGCACTGGATACGACGGAAATC-3'
miR-21	5'-GTCGTATCCAGTGCGTGTCGTGGAGTCGGCAATTGCACTGGATACGACTCAACAT-3'
miR-208	5'-GTCGTATCCAGTGCGTGTCGTGGAGTCGGCAATTGCACTGGATACGACGTATAAC-3'
miR-499	5'-GTCGTATCCAGTGCGTGTCGTGGAGTCGGCAATTGCACTGGATACGACAAACAT-3'
miR-214	5'-GTCGTATCCAGTGCGTGTCGTGGAGTCGGCAATTGCACTGGATACGACCTGCCT- 3'
miR-199b	5'-GTCGTATCCAGTGCGTGTCGTGGAGTCGGCAATTGCACTGGATACGACTAACCAA-3'
miR-26	5'-GTCGTATCCAGTGCGTGTCGTGGAGTCGGCAATTGCACTGGATACGACAGCCTAT-3'
miR-9	5'-GTCGTATCCAGTGCGTGTCGTGGAGTCGGCAATTGCACTGGATACGACTCATAC-3'
miR-27a	5'-GTCGTATCCAGTGCGTGTCGTGGAGTCGGCAATTGCACTGGATACGACGCGGAA-3'
miR-15b	5'-GTCGTATCCAGTGGGTGTCGTGGAGTCGGCAATTGCACTGGATACGACTGTAAAC-3'
U6	5'-CGCTTCACGAATTTGCGTGTCAT-3'
**PCR-primers**	
miR24-F	5'-GGGGTGGCTCAGTTCAGCA-3'
miR24-R	5'-CAGTGCGTGTCGTGGAGTC-3'
miR1-F	5'-GGGGTGGAATGTAAAGAAGTA-3'
miR1-R	5'-CGTGGAGTCGGCAATTGCA-3'
miR133a-F	5'-GGGTTTGGtCCCCTTCAA-3'
miR133a-R	5'-AGTGCGTGTCGTGGAGTC-3'
miR23a-F	5'-GGATCACATTGCCAGGGAT-3'
miR23a-R	5'-CAGTGCGTGTCGTGGAGT-3'
miR21-F	5'-GGGGTAGCTTATCAGACTG-3'
miR21-R	5'-TGGAGTCGGCAATTGCACTG-3'
miR208-F	5'-GCTTGAGCTTTTGGCCCG-3'
miR208-R	5'-CGTGGAGTCGGCAATTGC-3'
miR499-F	5'-GGGGTTAAGACTTGCAGTG-3‘
miR499-R	5'-CAGTGCGTGTCGTGGAGT-3'
miR214-F	5'-GGGGACAGCAGGCACAGAC-3'
miR214-R	5'-AGTGCGTGTCGTGGAGTCG-3'
miR199b-F	5'-GGGACAGTAGTCTGCACAT-3'
miR199b-R	5'-TGTCGTGGAGTCGGCAATT-3'
miR26-F	5'-GCGGTTCAAGTAATCCAGG-3'
miR26-R	5'-TGTCGTGGAGTCGGCAATT-3'
miR9-F	5'-GGGGTCTTTGGTTATCTAG-3'
miR9-R	5'-ATCCAGTGCGTGTCGTGGA-3'
miR27a-F	5'-GGGGTTCACAGTGGCTAA-3'
miR27a-R	5'-AGTGCGTGTCGTGGAGTC-3'
miR15b-F	5'-GGGTAGCAGCACATCATG-3'
miR15b-R	5'-TATCCAGTGCGTGTCGTG-3'
U6-F	5'-GCTTCGGCACATATACTAAAAT-3'
U6-R	5'-CGCTTCACGAATTTGCGTGTCAT-3'

### Knockdown of cardiac miR-21 expression using antagomir-21 and gene transfer of miR-21 mimic in vivo

Antagomirs are a novel class of chemically engineered antisense oligonucleotides. They differ from normal RNA by complete 2′-O-methylation of sugar, which are synthesized with 2′-OMe modified bases, phosphorothioate on the first 2 and last 4 bases, and a 3′ cholesterol modification through a hydroxyprolinol linkage [23]. Antagomirs are now used as a means to constitutively inhibit the activity of specific miRNAs [24]. In our current study, antagomir-21 was used to silence the cardiac endogenous miR-21 expression in vivo. The antagomir sequence complementary to mmu-miR-21 is 5′-gSaScagcccaucgacugcugSuSuSgS-Chol-3′. Chemically modified oligonucleotides 5′-gSuScaacuucagucagaaaagSgSuSaS-Chol-3′ were used as a negative control (scramble). Antagomir-21 was delivered into mouse myocardium using the local delivery method described in a recent study [25]. With the chest open as described above, 50 µL of phosphate-buffered saline (PBS) solution with antagomir-21 (80 mg/kg) was injected through a 26-gauge needle into the myocardium. Intramuscular injections were made in approximately 10 sites before coronary artery occlusion; this occlusion established infarction within the injected zone. In order to transfect the miR-21 mimic in vivo, with the chest open as described above, 50 µg in 100 µl of synthesized miR-21 (GenePharma Co. Ltd.) pretreated with lipofectamine 2000 (Invitrogen), was injected through a 26-gauge needle into the myocardium. After injection, the heart was placed back into the thoracic cavity, the air was expelled from the chest, and the chest was closed with sutures. 24 h later, cardiac IPost and I/R injury were performed in these mice and the heart tissues were isolated for experimental measurements.

### Infarct size measurement

Infarct size of the myocardium was measured as previously described [26]. 3 h after reperfusion, the ligature around the coronary artery was retied, and 1 ml of 5% Evans Blue dye was injected into the inferior vena cava. The heart was quickly excised after the dye was uniformly distributed. The ventricular tissues were dissected and kept overnight at -4°C. Frozen ventricles were sliced into 2 mm thick sections. The slices were incubated in 1% 2, 3, 5-triphenyltetrazolium chloride (TTC, Sigma-Aldrich) for 15 min at 37°C. Evans blue stained areas indicated the nonischemic area. Red parts in the heart, stained by TTC, represented for ischemic but viable tissue (area-at-risk). The pale white areas manifested infarcted myocardium. Areas of total left ventricular (LV), infarct size (INF) and area-at-risk (AAR) were measured digitally using Image Pro Plus software (version 6.0, Media Cybernetics). The percentage of the AAR/LV, INF/LV and INF/AAR was calculated respectively. Infarct size was expressed as a percentage of the INF/AAR.

### TUNEL assay

Apoptotic cardiomyocytes were detected using a terminal dUTP nick end-labeling (TUNEL) assay as previously described [27]. The TUNEL staining was detected using the in situ cell death detection kit (Roche) according to the manufacturer’s protocol. Sections were also co-stained with 4′, 6-diamidino-2-phenylindole (DAPI) (1:2 dilution, Invitrogen) for nuclei, and mouse monoclonal anti-sarcomeric α-actinin antibody (1:200 dilution, Sigma) conjugated with goat anti-mouse IgG-Alexa Fluor 594 (1:500 dilution, Invitrogen) for myocytes. Nuclei were counted in five microscopic fields from the midventricular section of each heart. The average of the TUNEL-positive nuclei ratio in at least five representative microscopic fields was calculated to compare the apoptosis ratio within the different groups.

### Left ventricular function evaluated by echocardiography

Echocardiograms were obtained at the end of 3 h of reperfusion. The mice were anesthetized with 2.5% (vol/vol) isoflurane and placed on the experimental platform. The coupling gel was applied to the mice chest. Transthoracic echocardiography was performed using a high-resolution in vivo ultrasound imaging system with a 40-MHz transducer (Panoview β1500, Cold Spring Biotech Corp.). Two-dimensional guided M-mode tracings were recorded from the parasternal long-axis view at the mid papillary muscle level. When the picture was stabilized, left ventricular end-diastolic dimensions (LVEDd), left ventricular end-systolic dimensions (LVESd), and heart rate (HR) were measured. Left ventricular ejection fraction (EF) and percentage fractional shortening (FS) were calculated with the accompanying software. The data were analyzed by a single observer blinded to mouse genotype.

### Western blot analysis

The total amount of protein was extracted from the left ventricular infarct region of mice for immunoblotting analysis. Briefly, the protein concentrations were determined with a bicinchoninic acid protein assay kit using bovine serum albumin as the standard. Equal amounts of protein (100 µg) were fractionated by SDS-PAGE and blotted to PVDF membrane (Millipore, Bedford, MA). The blots were blocked by 5% non-fat milk dissolved in PBS for 2 h, then probed overnight at 4°C with the following primary antibodies: PTEN (1:1000 dilution, Cell signaling Technology), Total Akt (1:200 dilution, Santa Cruz Biotechnology), p-Akt (1:1000 dilution, Cell signaling Technology), Bax (1:1000 dilution, Cell signaling Technology), Bcl-2 (1:1000 dilution, Cell signaling Technology), Caspase-3 (1:500 dilution, Cell signaling Technology) and anti-β-actin (1:200 dilution, Santa Cruz Biotechnology), all in 5% milk TBST. Membranes were washed three times, 15 min each time, with PBS containing 0.5% Tween 20 (PBS-T) and incubated with secondary antibody (1:8000 dilution, Alexa Fluor® 700 goat anti-mouse IgG (H+L) or Alexa Fluor® 800 goat anti-rabbit IgG (H+L), Invitrogen) in PBS at room temperature for 1 h. Western blot bands were captured by using the Odyssey Infrared Imaging System (LI-COR Biosciences, Lincoln, NE, USA) and quantified with Odyssey v1.2 software (LI-COR Biosciences, Lincoln, NE, USA) by measuring the band intensity (area×OD) in each group and normalizing to β-actin as an internal control. Unless otherwise stated, western blot experiments were repeated four times.

### Statistical analysis

All quantitative data are expressed as the mean±SEM and analysed by SPSS 13.0 software. Two-tailed unpaired Student’s t-tests and one-way ANOVA were used for statistical evaluation of the data. *P*<0.05 was considered as statistically significant.

## Results

### Mouse cardiac miRNA expression is regulated by IPost

**Figure 1:**
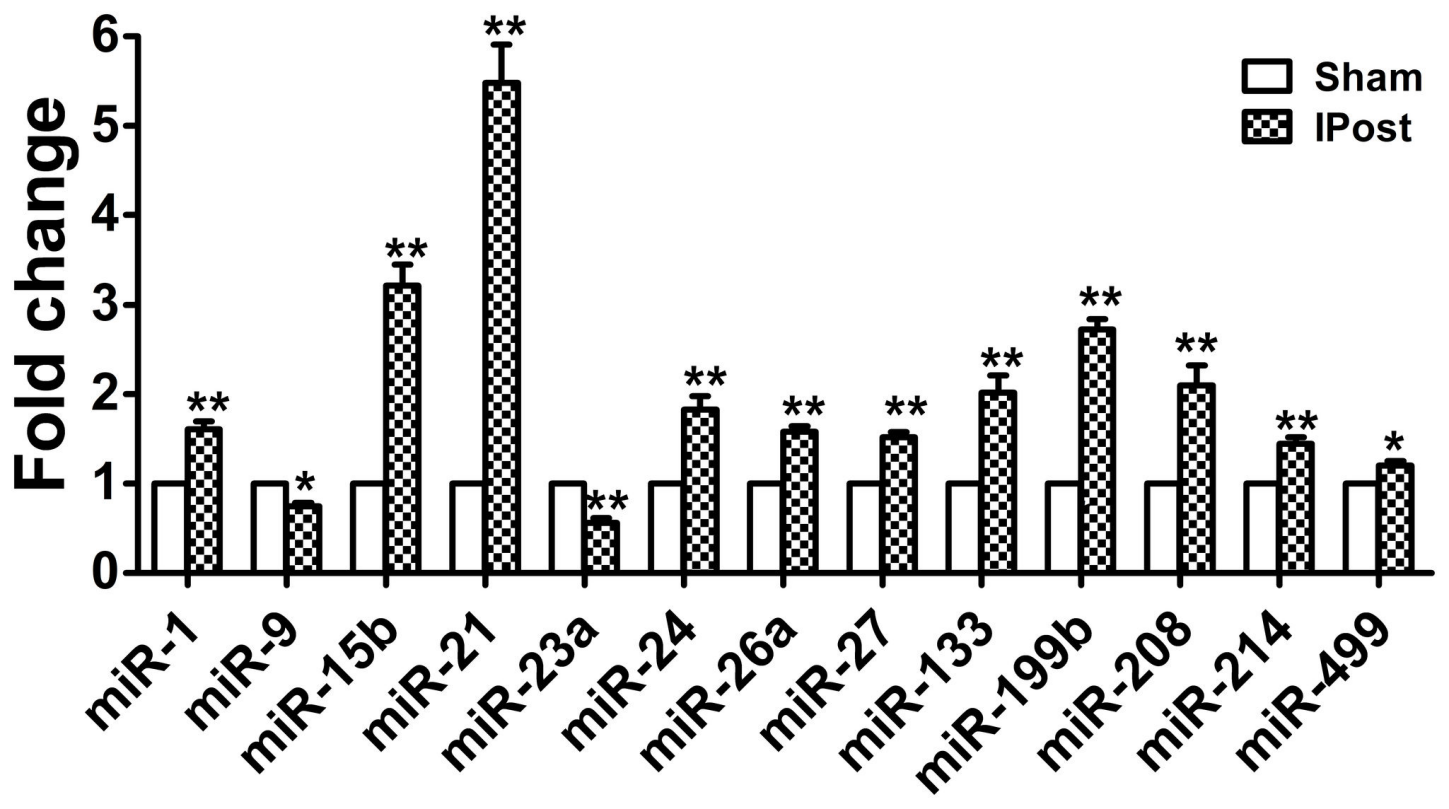
Aberrant expression of miRNAs in IPost mouse models. Verification of IPost-regulated miRNAs in mouse hearts. The expression of miRNAs in sham-opened mouse hearts (3 h) and in mouse hearts at 3 h after IPost was determined by qRT–PCR. IPost up-regulated miR-1, miR-15b, miR-21, miR-24, miR-26a, miR-27, miR-133a, miR-199a, miR-214, miR-208 and miR-499, while down-regulated miR-23a and miR-9 as compared with Sham group. Data are expressed as mean±SEM, n=5; ^*^ *P*<0.05, ^**^ *P*<0.01 compared with Sham group.

As previously reported, a collection of miRNAs were abnormally expressed in ischemic mouse hearts in response to I/R injury, such as miR-1, miR-9, miR-15b, miR-21, miR-23a, miR-24, miR-26a, miR-27, miR-133a, miR-199a, miR-208, miR-214 and miR-499 [20,21,28]. To investigate the potential involvement of these miRNAs in cardiac I/R injury, we used quantitative real-time RT-PCR analysis to determine miRNAs level in mouse hearts after IPost. Compared with sham group, the expressions of miR-1, miR-15b, miR-21, miR-24, miR-26a, miR-27, miR-133a, miR-199a, miR-214, miR-208 and miR-499 were increased in IPost hearts, while miR-9 and miR-23a were down-regulated in IPost models. Among these abnormally expressed miRNAs, we were excited to find that miR-21 was remarkably up-regulated in hearts by IPost and its expression was increased more than fivefold relative to sham group (Figure 1).

### Knockdown of cardiac miR-21 expression restrains IPost-mediated cardioprotection against I/R injury in vivo

**Figure 2:**
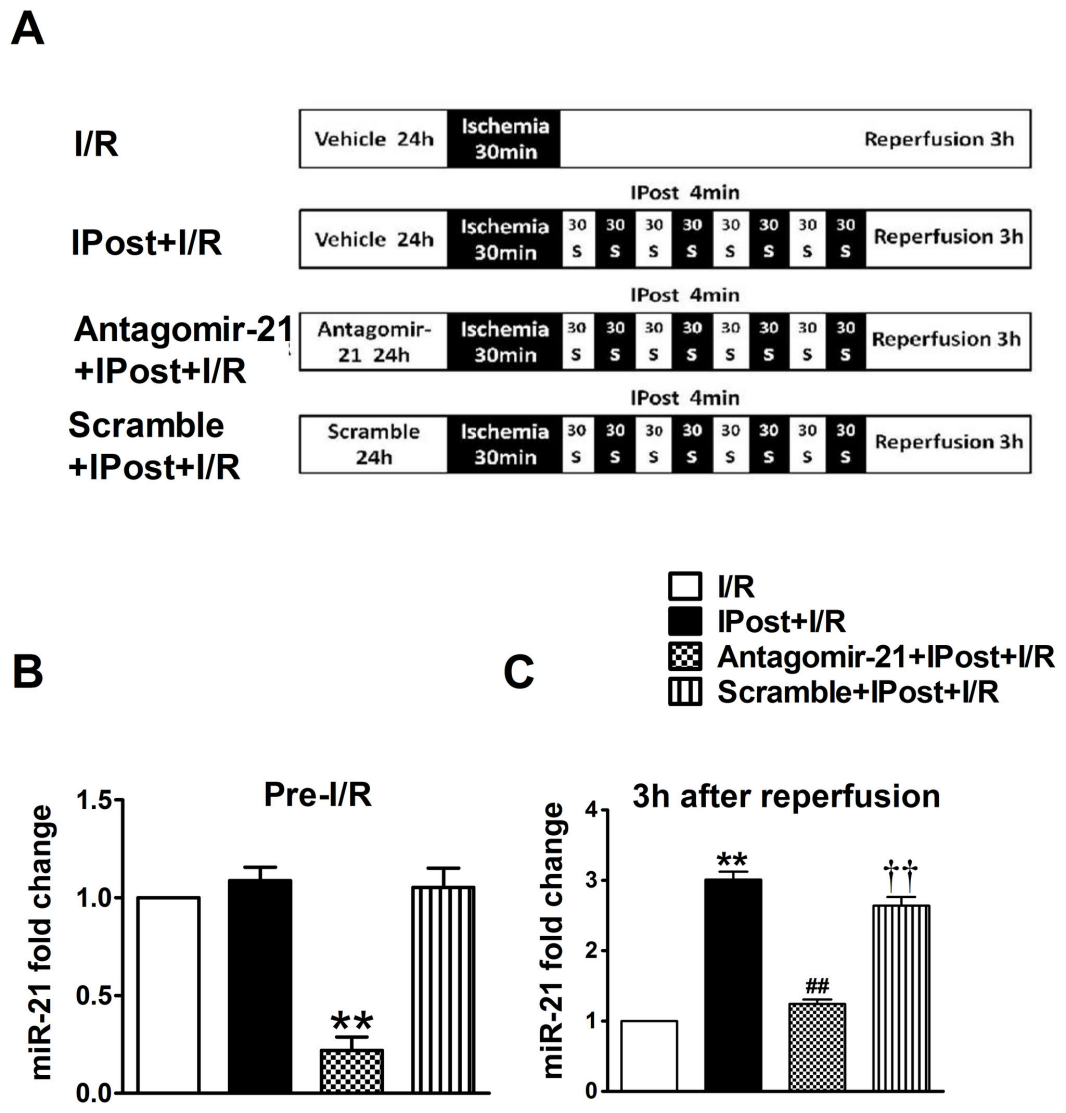
MiR-21 expression is blocked by its antagomir before and after 3 h of reperfusion in IPost-mediated cardiac protection. (A) Diagrammatic drawing showing the four groups of mice with different treatments. (B) miR-21 was markedly inhibited by antagomir-21 at the onset of I/R injury as determined by qRT–PCR. (C) IPost-induced upregulation of cardiac miR-21 was inhibited by antagomir-21 after 3 h of reperfusion as determined by qRT–PCR. Data are expressed as mean±SEM, n=5; ^**^ *P*<0.01 compared with I/R group; ^# #^ *P*<0.01 compared with IPost+I/R group; ^† †^ *P*<0.01 compared with Antagomir-21+IPost+ I/R group.

To determine the role of IPost-induced miR-21 in IPost-mediated cardiac protection, the mice were divided into the four groups as fellows (Figure 2A): I/R group; I/R+IPost group (I/R mice treated with IPost); Antagomir-21+IPost+I/R group (I/R mice treated with anagomir-21 and IPost); Scramble+IPost+I/R group (I/R mice treated with scramble and IPost).

To further explore the biological involvement of miR-21 in IPost-mediated cardiac protection, miR-21 expression was blocked by its antagomir. In our experiment, using the local delivery method described in Methods section, we delivered the antagomir-21 or scramble into the mouse myocardium at 24 h before IPost. As shown in Figure 2B, miR-21 could be down-regulated with antagomir-21 in vivo at the onset of I/R injury, while the scramble had not the same function. After 3 h of reperfusion, the expression of miR-21 in infarcted areas in IPost-pretreated mouse hearts was up-regulated relative to I/R group. Moreover, IPost-induced upregulation of cardiac miR-21 expression was successfully silenced by antagomir-21 as determined by qRT–PCR (Figure 2C).

**Figure 3:**
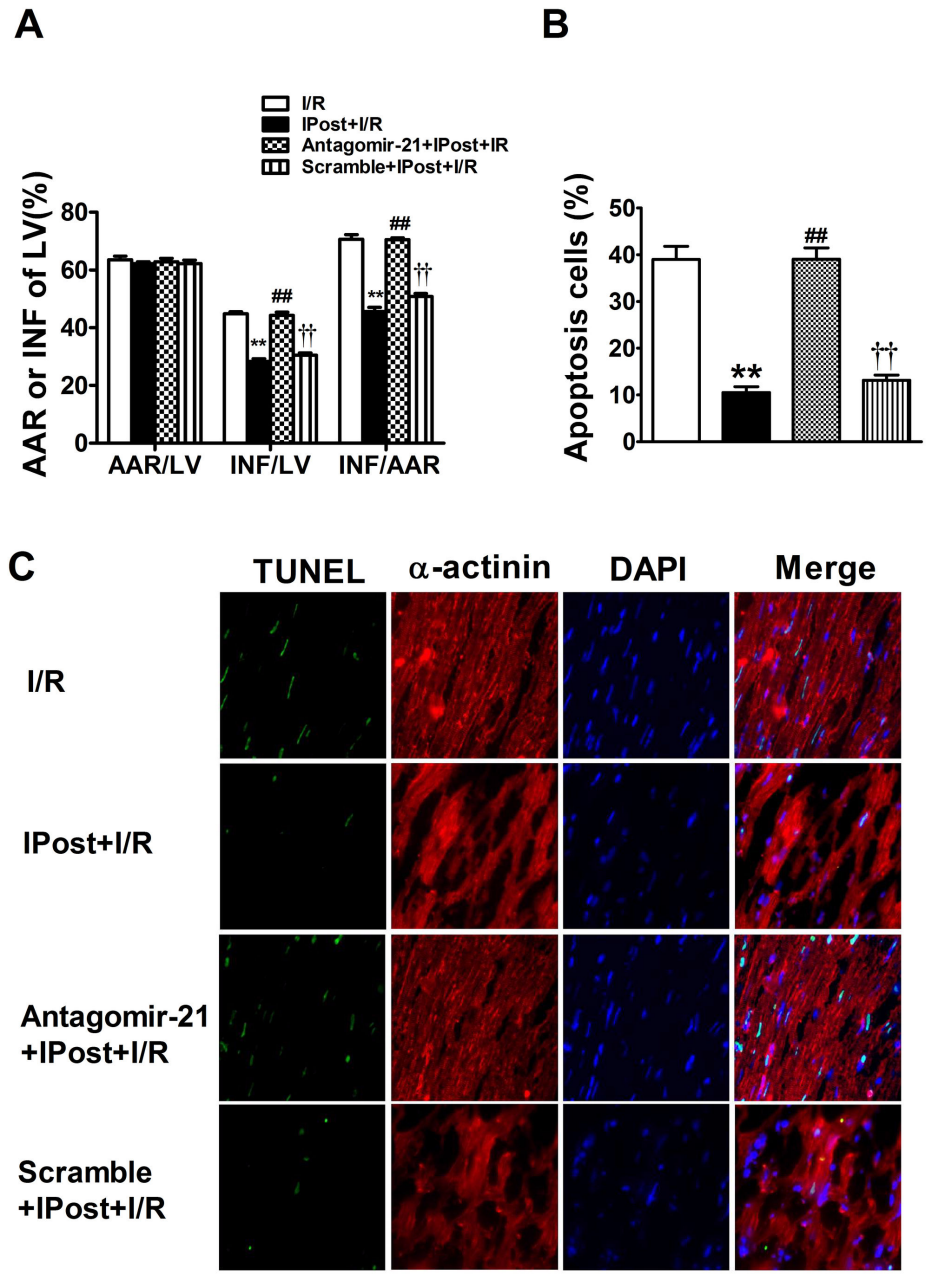
Knockdown of cardiac miR-21 expression abolishes IPost-mediated cardioprotection against I/R injury in vivo. (A) IPost-induced cardiac protection greatly reduced myocardial infarct size after 30 min myocardial ischemia followed by 3 h reperfusion. But antagomir-21 pre-treatment greatly increased myocardial infarct size. However, the region at risk was not significantly different among groups. Data are expressed as mean±SEM, n=5; ^**^ *P*<0.01 compared with I/R group, ^# #^ *P*<0.01 compared with IPost+I/R group; ^† †^ *P*<0.01 compared with Antagomir-21+IPost+ I/R group. (B) Knockdown of cardiac miR-21 expression inhibited IPost-induced anti-apoptotic effect on cardiac Cells in Vivo. Quantitative analysis of the apoptotic cells in heart sections. (C) Representative TUNEL and α-actinin stained photomicrographs of cardiac myocytes in heart sections from the four groups of mice with different treatments. Note: green colour was TUNEL staining representing apoptotic cells; red colour was the α-actinin staining representing cardiac myocytes; blue colour was the cell nucleus stained by DAPI. Original magnification: ×400. Data are expressed as mean± SEM, n=5; ^**^ *P*<0.01 compared with I/R group; ^# #^ *P*<0.01 compared with IPost+I/R group; ^† †^ *P*<0.01 compared with Antagomir-21+IPost+ I/R group.

The size of myocardial infarction was evaluated after 3 h of reperfusion. Histomorphometric analysis revealed that myocardial infarct size was significantly reduced by IPost relative to I/R group (Figure 3A). In addition, IPost-induced cardiac protection on myocardial infarct size was significantly attenuated in antagomir-21 group, but not in scramble pretreated animals. However, area-at-risk (AAR) was not significantly different among four groups (Figure 3A).

### IPost-mediated anti-apoptotic effect on cardiac cells in vivo is attenuated by knockdown of cardiac miR-21 with its antagomir

To further confirm the mechanism of IPost-upregulated miR-21-mediated cardiac protection in vivo, TUNEL staining was performed. Apoptosis was determined in heart sections from these four groups of mice by immunofluorescence with TUNEL staining. The myocytes nuclei were identified by immunofluorescence with DAPI staining. The cardiac myocytes were identified by immunofluorescence with α-actinin antibody staining. After 30 min ischemia and 3 h reperfusion, the percentage of TUNEL-positive cells in the zone of the previously ischemic area increased to (39.03±6.8%) (Figure 3B), while IPost could significantly decrease cardiac cell apoptosis (10.4±2.9%) (Figure 3B). However, compared with the IPost-pretreated group, the apoptosis of the cardiac cells in the IPost mice treated with anagomir-21 markedly increased to (36.7±5.9%) (Figure 3B), whereas the scramble (13.8±4.0%) (Figure 3B) had no significant effect on IPost-induced cardiac protection against apoptosis. Representative myocardial samples are shown in Figure 3C. Taken together, these data suggest that IPost-mediated miR-21 might protect mouse heart against I/R injury, probably through regulation of the cardiac cell apoptosis.

### The effect of IPost-induced miR-21 up-regulation on left ventricular function

**Figure 4:**
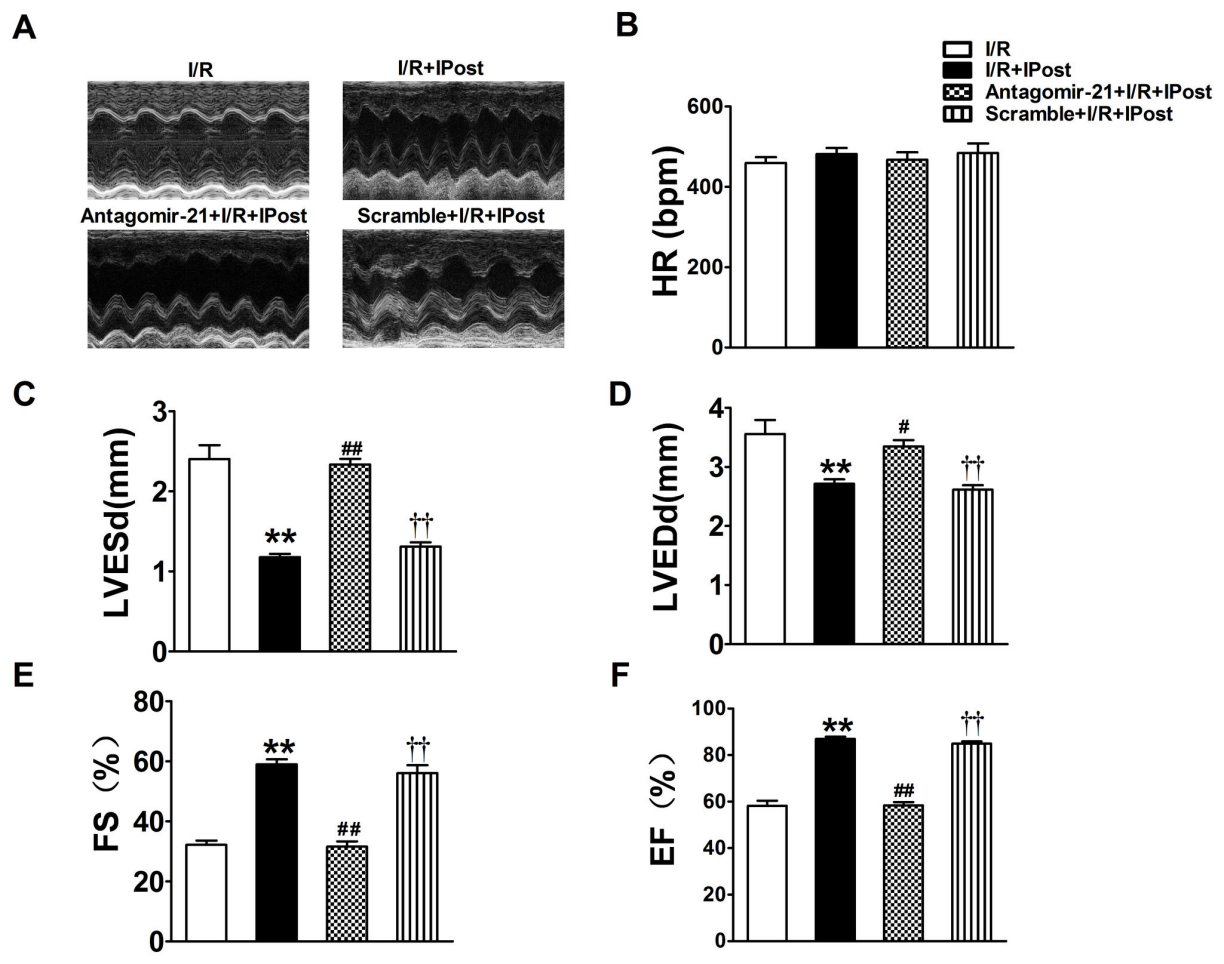
Echocardiographic assessment of left ventricular dimensions and function. Echocardiography was performed as described in Methods section (A) Representative M-mode echocardiographs from the four groups of mice with different treatments. (B) IPost treatment greatly improved left ventricular function after two weeks of reperfusion. But antagomir-21 pre-treatment exhibited significantly depressed cardiac function recovery during in vivo I/R mouse model, HR: heart rate; LVEDd: left ventricular end-diastolic dimensions; LVESd: left ventricular end-systolic dimensions; EF: ejection fraction; FS: percentage fractional shortening. Data are expressed as mean±SEM, n=5; ^**^ *P*<0.01 compared with I/R; ^#^ *P*<0.05, ^# #^ *P*<0.01 compared with IPost+I/R group; ^† †^ *P*<0.01 compared with Antagomir-21+IPost+ I/R group.

The reduced myocardial infarct size and cardiac cell apoptosis should have functional results on heart failure. To test these results, we used two-dimensional echocardiography and M-mode tracings to examine left ventricular function after 3 h of reperfusion. Heart rate, LVEDd, LVESd, EF and FS were evaluated by transthoracic echocardiography. Figure 4A shows M-mode echo tracings from individual mice representative of each experimental group. No significant differences were seen in heart rate in any group (Figure 4B). As expected, IPost significantly inhibited the increase of LVEDd and LVESd relative to I/R group (Figure 4C and D). In addition, IPost could obviously restore left ventricular FS and EF reduction in I/R mouse (Figure 4E and F). As shown in Figure 4C and D, the LVEDd and LVESd in antagomir-21 pre-treated animals were significantly increased relative to IPost-treated animals. Obviously, left ventricular FS and EF decreased after treatment with anagomir-21 (Figure 4E and F). However, the scramble had no effect on IPost-induced cardiac protection against heart failure. Together, these data disclose that IPost-induced cardiac functional recovery is associated with the increased level of miR-21.

### IPost-mediated miR-21 can signal through a PTEN/Akt axis in the cardiomyocytes, which is involved in anti-apoptotic effect on cardiac myocytes

**Figure 5:**
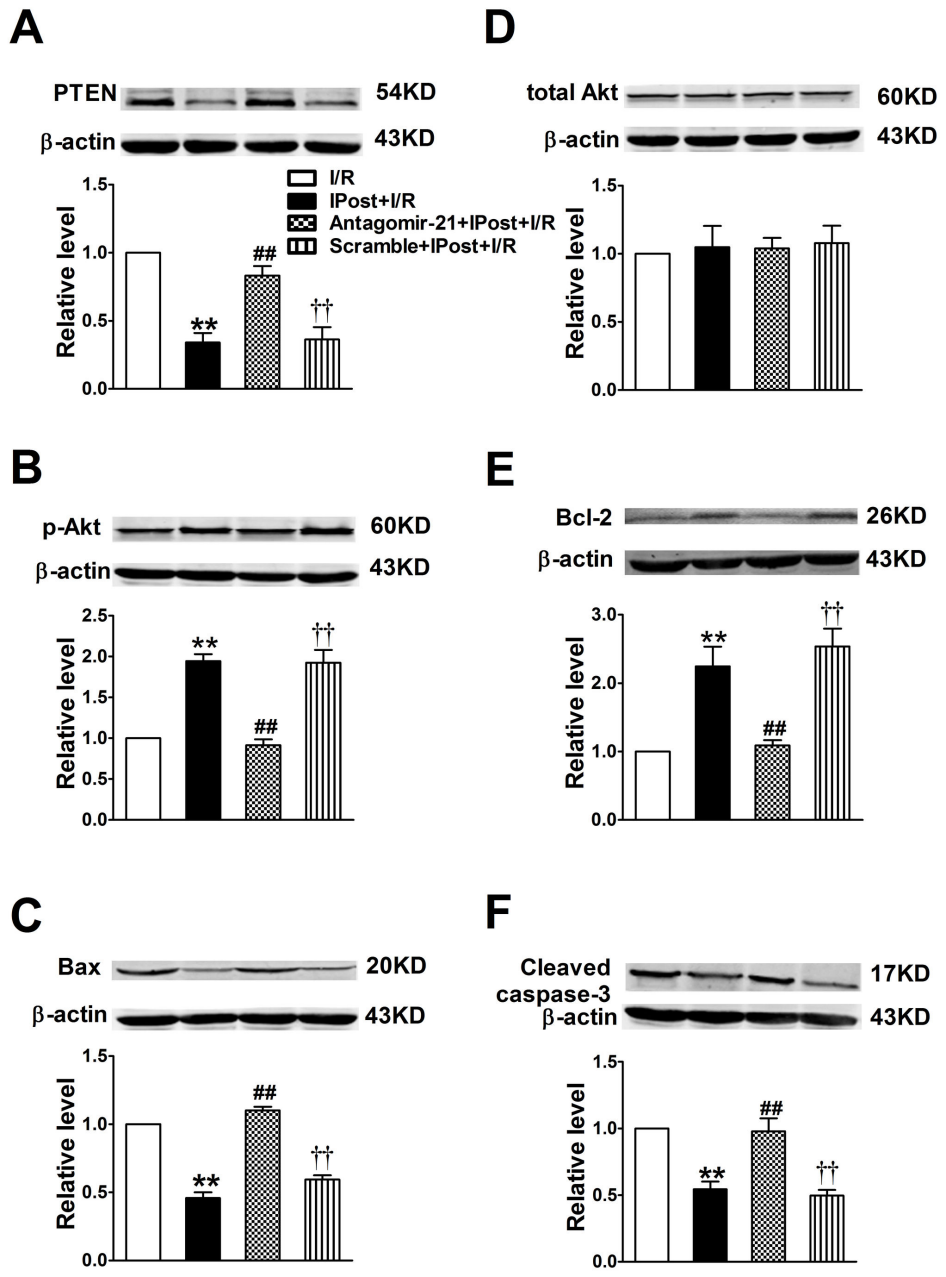
Modulation of PTEN p-Akt Total Akt Bcl-2 Bax and Cleaved caspase-3 protein expression in mouse cardiomyocyte by IPost and antagomir-21. Western blot and quantitative analysis of PTEN (A), Total Akt (B), p-Akt (C), Bcl-2 (D), Bax (E), Cleaved caspase-3 (F) in different groups. Data are expressed as mean±SEM, n=4; ^**^ *P*<0.01 compared with I/R; ^# #^ *P*<0.01 compared with IPost+I/R group; ^† †^ *P*<0.01 compared with Antagomir-21+IPost+ I/R group.

It has been reported that PTEN, not only is a vital antioncogene, but also is involved in regulating cellular survival, growth, hypertrophy and apoptosis [29]. Computational analysis indicates that PTEN is a potential target gene of miR-21 as described in recent study [30]. Moreover, PTEN is the major phosphatase that has been recently linked to the biology of myocardial I/R injury [31]. Hence, we sought to examine the significance of miR-21 in regulating PTEN/Akt signaling pathway in IPost mouse hearts. Our results indeed provided the evidence in support of this notion. As shown in Figure 5, IPost induced mild decrease in apoptosis-related proteins PTEN (Figure 5A), Bax (Figure 5E) and Caspase-3 (Figure 5F) levels compared with I/R group, accompanied by parallel up-regulation of miR-21 expression. However, IPost treatment did not affect total Akt levels relative to I/R group (Figure 5B). Furthermore, western blot analysis showed a significant up-regulation of p-Akt and Bcl-2 levels in IPost mouse hearts, and this up-regulation was also inhibited by antagomir-21 (Figure 5C and D). In summary, the results imply that IPost-mediated miR-21 can signal through a PTEN/Akt axis in the cardiomyocytes, which inhibits cardiac myocytes apoptosis during I/R injury.

### Inhibiting PI3K at time of reperfusion abrogates protection of miR-21 induced by IPost

**Figure 6:**
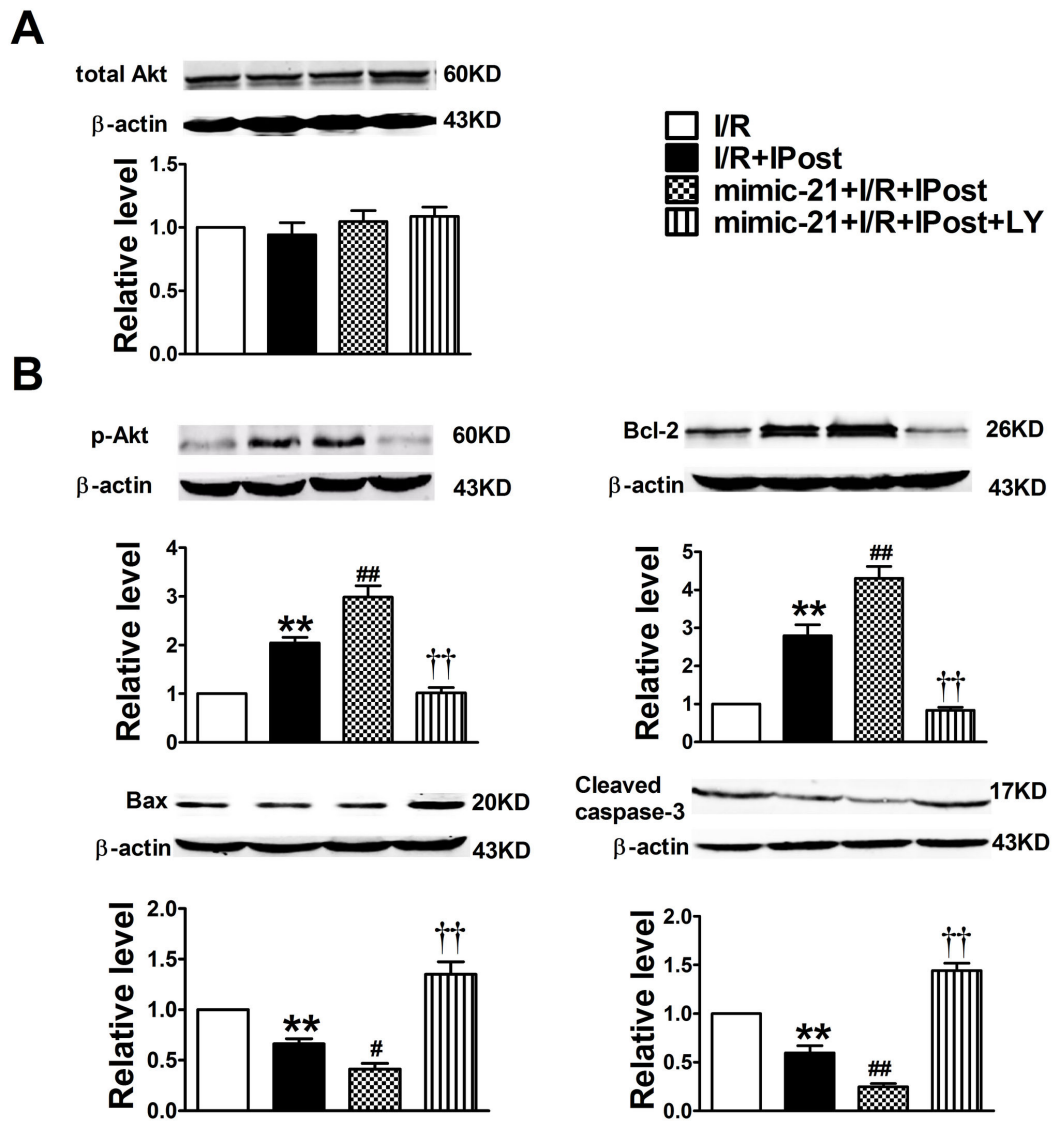
Modulation of total Akt and p-Akt protein expression in mouse cardiomyocyte by miR-21 and PI3K inhibitor LY294002. Western blot and quantitative analysis of Total Akt (A), p-Akt (B), Bcl-2 (C), Bax (D), Cleaved caspase-3 (E) in different groups. Note: LY is PI3K inhibitor LY294002. Data are expressed as mean±SEM, n=4; ^**^ *P*<0.01 compared with I/R; ^#^ *P*<0.05, ^# #^ *P*<0.01 compared with IPost+I/R group; ^† †^ *P*<0.01 compared with mimic-21+IPost+ I/R group.

In order to demonstrate the direct link between PTEN/Akt signaling pathway and miR-21 induced by IPost, we inhibited PI3K using the PI3K inhibitor LY294002, which was given for the first 15 minutes of reperfusion. The data in Figure 6A showed that there was no difference in total Akt expression in different conditions. But IPost could induce overexpression of p-Akt and Bcl-2 and down-regulation of Bax and Caspase-3 relative to I/R group (Figure 6B-6E). Moreover, overexpression of miR-21 could further increase expression of p-Akt and Bcl-2 (Figure 6B-6C), while decrease the expression of Bax and Caspase-3. However, PI3K inhibitor LY294002 could abrogate the cardioprotection of miR-21 against I/R injury in vivo (Figure 6B-6E).

**Figure 7:**
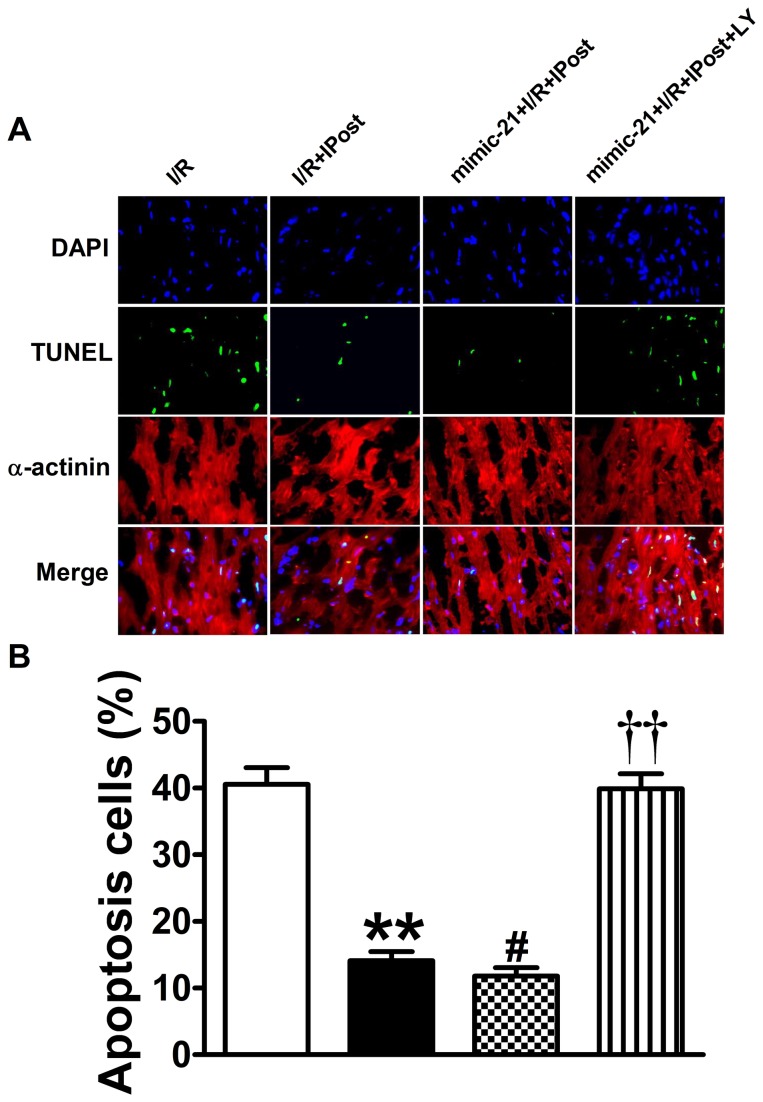
Inhibiting PI3K at time of reperfusion abrogated the anti-apoptotic role of miR-21 induced by IPost. (A) Representative TUNEL and α-actinin stained photomicrographs of cardiac myocytes in heart sections from the four groups of mice with different treatments. Note: LY was PI3K inhibitor LY294002; green colour was TUNEL staining representing apoptotic cells; red colour was the α-actinin staining representing cardiac myocytes; blue colour was the cell nucleus stained by DAPI. Original magnification: ×400. (B) Quantitative analysis of the apoptotic cells in heart sections. Data are expressed as mean±SEM, n=5; ^**^ *P*<0.01 compared with I/R group; ^#^ *P*<0.05 compared with IPost+I/R group; ^† †^ *P*<0.01 compared with mimic-21+IPost+I/R group.

In addition, the TUNEL staining also demonstrated that inhibiting PI3K at time of reperfusion abrogated the protection of miR-21 induced by IPost (Figure 7). The data in Figure 7 showed that overexpression of miR-21 could further decrease apoptosis relative to IPost group (from 14.08% to 11.80%, *P*<0.05; Figure 7B), while the PI3K inhibitor LY294002 could knockdown the anti-apoptotic role of miR-21 against I/R injury in vivo (from 11.80% to 39.90%, *P*<0.01; Figure 7B). Taken together, these results suggest that miR-21 induced by IPost acts directly on myocardium and induces cardioprotective effects through the activation of PTEN/Akt signaling pathway.

## Discussion

To our best knowledge, this is the first proof to demonstrate that miR-21 expression can be mediated by IPost, which plays an important role in protecting against cardiac I/R injury in mice models. The results presented here indicated that the potential signal pathway of miR-21 protection might be achieved by targeting PTEN/Akt signaling pathway.

In general, miRNAs are expressed at a constant level under physiological conditions. However, the endogenous expression levels of miRNAs are often altered in response to the physiological and pathological stimuli, tissue injury or milieu interieur disorders. By using quantitative real-time RT-PCR analysis, we found that a collection of miRNAs were regulated by IPost (Figure 1), such as miR-21, miR-15b, and miR-199b. The aberrant expression of miR-21 indicated that miR-21 was involved in the protective effect of cardiac IPost. In a recent study, Duan et al. found that there was no statistical significance in the expression of miR-21 in the remote ischemic preconditioning groups and IPost groups compared with the control group in the isolated rat heart model [32]. In different animal model, miR-21 expression induced by IPost is perhaps different, which possibly plays the role in ischemic cardioprotection via diverse pathways and causes various phenotypes, providing potential explanations for the currently divergent observations in our field.

Recent studies showed that ischemia induces profound changes in miRNAs expression in cardiac muscles [33,34]. More recently, the roles of miR-21 in cardiovascular disease have come into notice [34,35]. Yin et al. found that miR-21 had a protective role against I/R injury in an in vitro model [36]. Moreover, Cheng et al. demonstrated that IPC-mediated miR-21 had a protective role against I/R injury by reducing cardiac cell apoptosis via its target gene programmed cell death 4 (PDCD4) [20]. The direct proofs showing that miRNAs are involved in cardiac IPost were from recent one report [21], in which the expression of miR-133 and miR-1 were up-regulated by IPost. The current study for the first time demonstrated that miR-21 plays an important role in IPost-mediated protection against I/R injury. We found that miR-21 was up-regulated by IPost in mouse cardiac I/R injury model. But administration of anagomir-21 could silence the cardiac endogenous miR-21 expression in vivo. However, the similar phenomenon was not found in the scramble treatment group. In our research, we also observed that IPost played role in reducing INF of LV in mouse cardiac I/R injury model. In addition, we found that administration of anagomir-21, but not the scramble, could attenuate IPost-induced cardiac protection against I/R injury in the mouse hearts. Similarly, we found that antagomir-21-treated hearts exhibited significantly depressed cardiac function recovery after 3 h of reperfusion, evidenced by decreased left ventricular EF and FS but increased LVEDd and LVESd.

Accumulating evidences indicate that miRNAs are one kind of critical apoptotic regulator not only in tumor cells but also in heart cells. A recent study by Yang et al. has demonstrated that the muscle-specific miR-1 level is obviously increased in infarcted rat hearts where ischemic cardiomyocyte apoptosis plays an important role in cardiac ischemic injury [37]. Frank et al. has revealed that miR-20a is up-regulated in acute cardiac stress models and attenuates hypoxia-mediated cardiomyocyte apoptosis [38]. A recent elegant research by Wang et al. has showed that miR-494 targets both anti-apoptotic and pro-apoptotic proteins and plays an important role in protecting against I/R-mediated cardiac injury [17]. MiR-21 is a highly expressed miRNA in cardiovascular system, which has also been implicated in cardiomyocyte apoptosis [34,36]. A recent study has shown that miR-21 was upregulated after IPC and miR-21 was thought to be involved in miRNA-induced cardioprotection [36].

It is noteworthy that PTEN is a key molecule in the development of many cardiovascular diseases because PTEN is widely expressed in endothelial cells, vascular smooth muscle cells, cardiac muscle cells, and fibroblasts where it modulates hypertrophy, contractility, cell survival/apoptosis and metabolism via its target molecules, phosphoinositide- 3kinases (PI3Ks) and Akt [39]. PTEN is the current identified target genes of miR-21 which is involved in miR-21-mediated cardiovascular effects [35]. Furthermore, PTEN activity is lowered after IPC and restored when the protective role of preconditioning decays [40]. So we selected PTEN as the potential target protein of miR-21 to see whether miR-21 was involved in the IPost-mediated anti-apoptotic effects on cardiomyocyte apoptosis. Our data presented in current study indicated that IPost inhibited the expression of PTEN during I/R injury, accompanied by parallel up-regulation of miR-21 expression. More exciting, we observed that knockdown of endogenous miR-21 expression with antagomir-21 increased sensitivity to I/R-triggered cell death. In addition, PTEN expression was up-regulated by knockdown of endogenous miR-21 expression in vivo using its antagomir during I/R injury. After transferring antagomir-21 or the scramble into myocardium in vivo, we found that antagomir-21, but not the scramble, could attenuate IPost-induced cardiac protection against apoptosis induced by simulated I/R injury. Findings of this study firstly present evidence demonstrating that in cardiocytes, IPost-mediated miR-21 negatively regulates PTEN expression during I/R injury. Furthermore, we found that IPost increased Bcl-2 protein level, and attenuated the expression of Bax and Caspase-3 proteins in mouse I/R injury heart. However, after transferring antagomir-21 into myocardium to silence the endogenous miR-21 in vivo, the anti-apoptotic effects of IPost were attenuated markedly in vivo. We found that antagomir-21 promoted the expression of Bax and Caspase-3 proteins, and decreased Bcl-2 protein level. Furthermore, inhibiting PI3K at time of reperfusion abrogated cardiac protection of miR-21 induced by IPost. Taken together, these data clearly demonstrate that miR-21, as an anti-apoptotic miRNA, plays an anti-apoptotic role via activation of the PTEN/Akt signaling pathway in cardiac IPost model. It should be noted that IPost-mediated cardiac protection against myocardial I/R injury was partially inhibited by knockdown of cardiac miR-21, indicating that miR-21 may be an important target for the development of novel therapeutic strategies for protection against ischemic insults.

In summary, our data suggest that miR-21 plays an important role in IPost-induced protective effects against myocardial I/R injury. Up-regulating of endogenous miR-21 induced by IPost is able to alleviate I/R-induced cardiomyocyte apoptosis of the infarct area in mouse heart and the potential mechanism is involved in regulation of PTEN/Akt signaling pathway. This study indicates that IPost-regulated miR-21 may be a promising intervention in the management of ischemic heart diseases. It should be pointed out that our studies were performed in animal models and the experimental results may not be extrapolated directly to humans. However, the findings open the door for further studies to investigate whether the roles of miR-21 induced by IPost also operate in the clinical practice.
